# The Treatment of Amaurosis and Amblyopia by Strychnia

**Published:** 1873-02

**Authors:** 


### THE TREATMENT OF AMAUROSIS AND AMBLYOPIA BY STRYCHNIA.

Our attention is called, in a pamphlet by Dr. Albrecht Nagel, to the hypodermic use of strychnia. The method has been already known for a sufficiently long time to permit of comparative trials by other observers, and their results in the main agree with the statements of Professor Nagel. It is understood that a large class of cases of amaurosis and amblyopia remain incurable, viz: such as depend on extreme textural change; but for a considerable contingent, which by other methods are scarcely at all relieved, the hypodermic use of strychnia has been greatly efficacious. Our present methods of diagnosis do not show us the minute structure of the optic nerve and ratina, and we are therefore unable to tell, in a case of atrophy, just how far the fibres or ultimate elements are impaired; hence, the trial of any remedy must be attended in all cases with a degree of uncertainty.

But what the method of Professor Nagel has done is a positive gain. It is as follows: He injects into the temple a moderate dose of nitrate of strychnia, say from 1-36 to 1-16 grain every day for a week. He expects the effect on vision to be produced within an hour. This may be either in extension of the limits of a contracted field, or in the increase of direct vision. If this result is not obtained within a few days, the dose is increased, but not above 1-12 of a grain. If no good effect, the case is regarded as unsuited for this treatment. The diseases for which this treatment has been greatly useful have been, a boy with imperfect amaurosis of one eye, with concentric limitation of the field, and no opthalmoscopic appearances; after one injection of 1-32 grain nitrate strychnia, vision rose in half an hour from $\frac{15}{LXX}$ to $\frac{15}{XV}$, and the field was a little enlarged. The improvement was maintained for two days, when another injection of 1-27 grain added a little more to the area of the field, and improvement afterward went on spontaneously to perfect cure.

Another case of amaurosis, following measles, complicated with head-symptoms, was treated with perfect success. The interior of the eyes exhibited no lesions. Slow improvement of sight was taking place under general tonics and hygiene; but, to hasten the progress, strychnia was tried. The first injection of 1-53 grain had no effect; a second, of 1-40 grain, brought vision from less than $\frac{1}{CC}$ to $\frac{1}{XX}$. In a month after, four injections, v. = $\frac{15}{XV}$.

Another case, a child of three years, in whom the state of the optic nerve was almost perfectly normal although blindness was complete, proved obdurate to strychnia. No treatment did any good.

Other classes of cases successfully handled were asthenopia of the accommodative kind, amblyopia from disuse, traumatic amaurosis, etc. A case of embolus of the central artery of the retina is contributed by Professor Becker, in which the striking gradations of improvement in the field are shown by diagrams. The case looks more like one of neuritis, with consecutive thrombosis, than pure embolus. At the beginning, the arteries were extremely small, and the nerve a little large, the region of the macula infiltrated. At the close of a month, vision was $\frac{20}{XX}$, but the field was still limited upward and outward. The infiltration about the macula had disappeared, but the nerve showed appearances of atrophy. Such are some of the important cases discussed in this pamphlet, and the treatment is well worth imitating. The use of strychnia by the stomach, and sprinkled on a blistered surface, does not have the same effect as when injected. This fact, moreover, has been substantiated in treating other diseases of the nervous system, so that the recommendations of Professor Nagel are well supported.

It is proper to say that Professor Nagel gives a caution against the excessive use of the remedy, as liable to defeat the object of treatment by irritating too severely the nerve-tissue, even though there may be no evident symptoms of poisoning.—*Dr. Henry D. Noyes, in New York Medical Record.*

